# Caloxanthones O and P: Two New Prenylated Xanthones from *Calophyllum inophyllum*

**DOI:** 10.3390/molecules15020606

**Published:** 2010-01-27

**Authors:** Hao-Fu Dai, Yan-Bo Zeng, Qi Xiao, Zhuang Han, You-Xing Zhao, Wen-Li Mei

**Affiliations:** 1 Key Laboratory of Tropical Crop Biotechnology, Ministry of Agriculture, Institute of Tropical Bioscience and Biotechnology, Chinese Academy of Tropical Agricultural Sciences, Haikou 571101, Hainan, China; E-Mails: hfdai2001@yahoo.com.cn (H-F.D.); zengyanbo@163.com (Y-B.Z.); qixiao78wm@163.com (Q.X.); hanzone@yahoo.cn (Z.H.); 2 International Joint Laboratory for Research and Development of Tropical Medicinal Plants, Chinese Academy of Tropical Agricultural Sciences, Haikou 571101, China; 3 State Key Laboratory of Phytochemistry and Plant Resources in West China, Kunming Institute of Botany, Chinese Academy of Sciences, Kunming 650204, Yunnan, China; E-Mail: xyzhao@mail.kab.ac.cn (Y-X.Z.)

**Keywords:** *Calophyllum inophyllum*, caloxanthone O, caloxanthone P, cytotoxic activity, prenylated xanthones

## Abstract

Chemical investigation of the EtOH extract of the twigs of *Calophyllum* *inophyllum* collected in Hainan Province of China resulted in the isolation of two new prenylated xanthones, caloxanthone O (**1**) and caloxanthone P (**2**). Their structures were elucidated by a study of their physical and spectral data. Compound **1** exhibited cytotoxicity against human gastric cancer cell line (SGC-7901), with an IC_50_ value of 22.4 *μ*g mL^−1^.

## Introduction

*Calophyllum inophyllum* Linn, which belongs to the family Clusiaceae, is an evergreen shrub widely distributed in tropical areas. It is used in traditional Chinese folk medicine for the treatment of eye diseases, wounds, rheumatism and inflammations [1,2]. The chemical literature reflects the existence of wide variety of natural products in this plant, such as pyranocoumarins [3,4,5,6,7,8,9], xanthones [4,5,8], triterpenes [9,10], and flavonoids [11], which possessed various bioactivities such as anti-HIV-1 [3], anti-microbial [9], and cytotoxic activities [9,12]. Previously, we isolated a new cytotoxic prenylated xanthone from the twigs of *C. inophyllum* collected in Hainan Province of China [12]. In the continuous search for bioactive constituents, two new prenylated xanthones, named caloxanthones O (**1**) and P (**2**) were obtained. Compound **1** exhibited cytotoxicity against human gastric cancer cell line. The present paper discusses their structural elucidation and cytotoxicity.

## Results and Discussion

Compound **1**, was obtained as a yellow powder that reacted positively to the Gibbs and FeCl_3_ reagent, indicating the presence of phenolic groups. Its HR-ESI-MS spectrum showed the quasi-molecular [M+Na]^+^ ion peak at *m*/*z* 467.1681 (calc. 467.1676), corresponding to the molecular formula C_24_H_28_O_8_. This formula could also be validated through its ^1^H-NMR, ^13^C-NMR and DEPT data. The IR spectrum displayed free hydroxyl (3,560 cm^−1^), chelated hydroxyl (3,160 cm^−1^), conjugated carbonyl (1684 cm^−1^), and aromatic ring (1,589, 1,540 cm^−1^) absorptions. These data, together with those obtained from the UV spectrum [λ_max_ (MeOH) 217, 222, 252, 282, 321 nm] were consistent with the presence of a xanthone skeleton [4, 5]. In the ^1^H-NMR spectrum, a chelated hydroxyl group *δ*_H_ 13.73 (s, 1H), two isolated aromatic protons *δ*_H_ 6.19 (s, 1H), 6.78 (s, 1H) and one methoxyl (*δ*_H_ 3.84) were observed. The ^1^H-NMR spectrum of **1** also showed two methyl singlets (*δ*_H_ 1.25 and *δ*_H_ 1.55), a methyl doublet (*δ*_H_ 1.36, *J* = 6.5 Hz) and one-proton quartet (*δ*_H_ 4.56, *J* = 6.2 Hz), which suggested the presence of an α,α,β-trimethyldihydrofuran ring [4]. Furthermore, two methyl singlets (*δ*_H_ 1.15, *δ*_H_ 1.17), an oxymethine proton (*δ*_H_ 3.41, 1H, dd, *J* = 6.3, 9.6 Hz ), and two methylene protons (*δ*_H_ 2.62 and 3.78, each 1H) were observed in the ^1^H-NMR spectrum, which indicated the presence of a C_5_ unit characterized as a 2,3-dihydroxy-3-methylbutyl chain in **1** [13]. Assignment of the ^13^C-NMR spectral data are shown in Table 1. A combination of the ^1^H-^1^H COSY and HMQC experiments permitted the assignment of all the protonated carbons. It remained to establish the positions of the substituents on the xanthone skeleton. In the HMBC spectrum (Figure 2), the proton of chelated hydroxyl group 1-OH (*δ*_H_ 13.73) was correlated to three carbons C-9a (*δ*_C_ 102.7), C-2 (*δ*_C_ 92.9), and C-1 (*δ*_C_ 163.5), which suggested that the chelated hydroxyl group was located at C-1. Aromatic carbons with an oxygen function were observed at C-1 (*δ*_C_ 163.5), C-3 (*δ*_C_ 164.9) and C-4a (*δ*_C_ 151.3) in the ^13^C-NMR spectrum, which indicated that this aromatic ring was a phloroglucinol ring [14]. Therefore, the α,α,β-trimethyldihydrofuran ring was clearly fused at C-4 through an oxygen at C-3 position. This result was further confirmed by the long-range correlations of H-12 (*δ*_H_ 1.25) and H-13 (*δ*_H_ 1.55) with C-4 (*δ*_C_ 112.0) and H-14 (*δ*_H_ 4.56) with C-3 (*δ*_C_ 164.9) in the HMBC spectrum. The positions of the 2,3-dihydroxy-3-methylbutyl chain and the remaining phenolic hydroxyl group were established as follows. In the HMBC spectrum (Figure 2), the methylene protons H_2_-16 (*δ*_H_ 3.78 and *δ*_H_ 2.62) were correlated to three aromatic carbons C-8a (*δ*_C_ 110.8), C-7 (*δ*_C_ 117.7), and C-8 (*δ*_C_ 139.4). The latter resonance at C-8 also gave cross-peak with the oxymethine proton H-17 (*δ*_H_ 3.41). These results demonstrated clearly that the 2,3-dihydroxy-3-methylbutyl moiety was located at C-8. The cross-peaks from the aromatic proton (*δ*_H_ 6.78) to C-16 (*δ*_C_ 36.7), C-8a (*δ*_C_ 110.8), and C-5 (*δ*_C_ 132.8) in the HMBC spectrum indicated that the aromatic proton (*δ*_H_ 6.78) was assigned at C-7 position. The methoxyl group was deduced to be located at C-5 position by ROESY experiment revealing the cross-peak from the 5-OMe (*δ*_H_ 3.84) to H-13 (*δ*_H_ 1.55) (Figure 2). The downfield shifts of C-6 (*δ*_C_ 155.3) and C-18 (*δ*_C_ 71.9) revealed that each carbon should be connected to a hydroxyl group. The configuration at C-17 has not been determined in this study because the amount of compound **1** is too low. On the basis of the above results, the structure of compound **1** was thus elucidated and named caloxanthone O.

**Figure 1:**
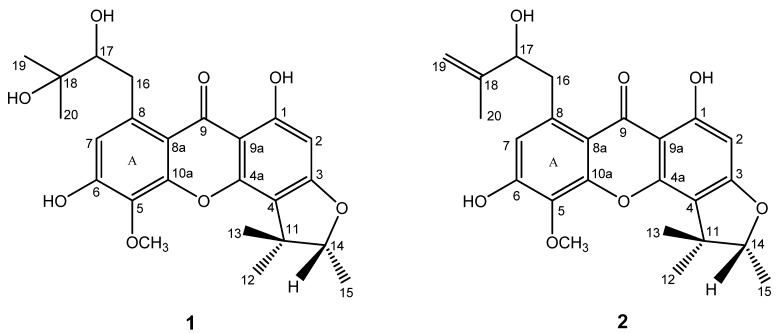
Structures of compounds **1** and **2**.

**Figure 2:**
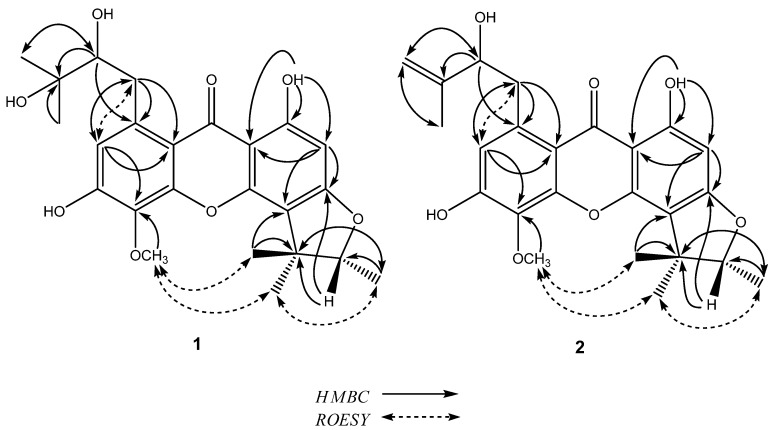
Key HMBC and ROESY correlations of compounds **1** and **2**.

Compound **2**, was obtained as yellow powder and reacted positively to the Gibbs and FeCl_3_ reagent. The [M+Na]^+^ at *m/z* 449.1576 in the HR-ESI-MS spectrum corresponds to the molecular formula C_24_H_26_O_7_ (calc. 449.1571). The IR and UV spectra were suggestive of a xanthone derivative. The ^1^H- and ^13^C-NMR were closely related to those of **1**, except for the substitution of C_5_ unit side chain in ring A. The ^1^H-NMR spectrum of **2** showed a methyl singlet (*δ*_H_ 1.86), an olefinic methylene (*δ*_H_ 4.77 and 4.96, each 1H), an oxymethine (*δ*_H_ 4.35, dd, *J* = 8.8, 3.6 Hz) and a methylene (*δ*_H_ 3.02, dd, *J* = 12.6, 3.6 Hz and *δ*_H_ 3.76, dd, *J* = 12.6, 3.6 Hz), which established a 2-hydroxy-3-methylbut-3-enyl group. The positions of the substitutions in **2** were established by HMBC and ROESY spectra (Figure 2). The configuration at C-17 has not been determined in this study because the amount of compound **2** is too low. From the above evidence, the structure of the compound **2** was thus confirmed, and given the trivial name caloxanthone P.

Compounds **1** and **2** were evaluated for their cytotoxic activity against human gastric cancer cell line (SGC-7901) using the MTT method. Compound **1** showed cytotoxic activity against the SGC-7901 cell line with the IC_50_ value of 22.4 *μ*g mL^−1^, while compound **2** was inactive (IC_50_ > 100 *μ*g mL^−1^).

## Experimental

### General

Melting points were obtained on a Beijing Taike X-5 stage apparatus and are uncorrected. Optical rotation was recorded using a Rudolph Autopol III polarimeter (Rudolph Research Analytical, NJ, USA). The UV spectra were measured on a Shimadzu UV-2550 spectrometer. The IR spectra were obtained on a Nicolet 380 FT-IR instrument, as KBr pellets. The NMR spectra were recorded on a Bruker AV-400 spectrometer, using TMS as an internal standard. The HRESIMS spectra were measured with an API QSTAR Pulsar mass spectrometer. Column chromatography was performed with silica gel (Marine Chemical Industry Factory, Qingdao, China) and Sephadex LH-20 (Merck). TLC was preformed with silica gel GF254 (Marine Chemical Industry Factory, Qingdao, China).

### Plant Material

The twigs of *Calophyllum inophyllum* L. were collected in Wenchang county, Hainan Province, China in May 2006, the plant was identified by Associate Professor Zheng-Fu Dai of the Institute of Tropical Bioscience and Biotechnology, Chinese Academy of Tropical Agricultural Sciences, where a voucher specimen (No. 20060508) of *C. inophyllum* was deposited.

### Extraction and Isolation

The dried and crushed twigs of *C. inophyllum* (19.9 kg) were extracted with 95% EtOH (70 L) three times at room temperature. After removal of EtOH by evaporation, the EtOH extract was suspended in water (6.0 L) and successively partitioned with petroleum ether (4 L) three times to give a Petro-soluble extract (290.0 g) and an aqueous residue. The Petro-soluble extract (290.0 g) was applied to a silica gel (200-300 mesh) column packed in CHCl_3_. The column was then eluted in gradient elution with CHCl_3_-acetone (1:0, 20:1, 10:1, 5:1, 2:1, 0:1) to afford 16 fractions. The active fraction (fraction 16, 44.0 g) was then subjected to repeated column chromatography over silica gel using Pet-EtOAc as eluent and further separated by column chromatography over Sephadex LH-20 using 95% EtOH as eluent to afford **1** (5.6 mg). The 95% MeOH fraction (245.0 g) was subjected to vacuum liquid chromatography (VLC) over silica gel, eluting with gradient elution CHCl_3_-MeOH to afford 10 fractions. The active fraction (fraction 3, 5.7 g) was then subjected to repeated column chromatography over silica gel using Pet-acetone as eluent and further separated by column chromatography over Sephadex LH-20 using 95% EtOH as eluent to afford **2** (6.2 mg).

*Caloxanthone O:* Yellow powder, M.p. 228– 230 ºC. [*α*]${}_{D}^{25}$ = + 32.0 (*c* = 0.50, MeOH). UV (MeOH): λ_max_ (log *ε*_max_): 217 (1.25), 222 (sh), 252 (0.51), 282 (0.32), 321 (0.28) nm. IR (KBr): ν = 3560, 3160, 2314, 1684, 1589, 1540, 1419, 1384, 843 cm^−1^. HRMS ((+)-ESI): *m/z* = 467.1681 (calcd. 467.1676 for C_24_H_28_O_8_Na, [M + Na]^+^). ^1^H and ^13^C-NMR: see Table 1.

*Caloxanthone P:* Yellow powder, M.p. 203 − 204 ºC. [*α*]${}_{D}^{25}$ = + 56.0 (*c* = 0.50, MeOH). UV (MeOH): λ_max_ (log *ε*_max_): 219 (1.95), 258 (2.17), 281 (1.36), 325 (1.47) nm. IR (KBr): ν = 3556, 3473, 3414, 2414, 2230, 1725, 1619, 1585, 1343, 1276, 1048, 907, 861, 830 cm^−1^. HRMS ((+)-ESI): *m/z* = 449.1576 (calcd. 449.1571 for C_24_H_26_O_7_Na, [M + Na]^+^). ^1^H and ^13^C-NMR: see Table 1.

### Bioassay 

The 3-(4,5-dimethylthiazol-2-yl)-2,5-diphenyltetrazolium bromide (MTT) assay was performed according to the previously reported method [15]. The inhibition rates (IR%) were calculated using OD mean values from IR% = (OD_control_ − OD_sample_)/OD_control_. The IC_50_ value, which was defined as the concentration of sample needed to reduce 50% of absorbance relative to the vehicle-treated control, was determined using the Bliss method. The same experiment was repeated independently three times to obtain a mean IC_50_ and its standard deviation.

## 4. Conclusions

It was reported that a wide variety of bioactive natural products exist in the plant *Calophyllum inophyllum*, such as pyranocoumarins, triterpenes, and flavonoids, together with xanthones, especially prenylated xanthones. In our continuous search for bioactive constituents, two new prenylated xanthones, named caloxanthones O (1) and P (2) were obtained. Compound 1 showed cytotoxic activity against the SGC-7901 cell line with the IC_50_ value of 22.4 *μ*g mL^−1^, while compound 2 was inactive (IC_50_ > 100 *μ*g mL^−1^).

## Figures and Tables

**Table 1:** ^1^H- and ^13^C-NMR data of **1** and **2**. (^1^H at 400 and ^13^C at 100 MHz; *J* in Hz).

No.	1^a^		2^b^	
	*δ_C_*	*δ*_H_	*δ_C_*	*δ*_H_
1	163.5 (*s*)		166.5 (*s*)	
2	92.9 (*d*)	6.19 (1H, *s*)	95.1 (*d*)	6.13 (1H, *s*)
3	164.9 (*s*)		167.7 (*s*)	
4	112.0 (*s*)		114.1 (*s*)	
5	132.8 (*s*)		135.4 (*s*)	
6	155.3 (*s*)		153.8 (*s*)	
7	117.7 (*d*)	6.78 (1H, *s*)	119.5 (*d*)	6.83 (1H, *s*)
8	139.4 (*s*)		140.6 (*s*)	
9	181.5 (*s*)		184.2 (*s*)	
4a	151.3 (*s*)		153.9 (*s*)	
8a	110.8 (*s*)		113.8 (*s*)	
9a	102.7 (*s*)		105.3 (*s*)	
10a	151.3 (*s*)		156.8 (*s*)	
11	43.0 (s)		45.5 (*s*)	
12	21.1 (*q*)	1.25 (3H, *s*)	22.7 (*q*)	1.33 (3H, *s*)
13	25.0 (*q*)	1.55 (3H, *s*)	26.9 (*q*)	1.63 (3H, *s*)
14	90.3 (*d*)	4.56 (1H, *q*, 6.2 Hz)	92.6 (*d*)	4.57 (1H, *q*, 6.5 Hz)
15	13.9 (*q*)	1.36 (3H, *d*, 6.5 Hz)	15.5 (*q*)	1.41 (3H, *d*, 6.6 Hz)
16	36.7 (*t*)	3.78 (1H, *d*, 12.8 Hz, H-16a) 2.62 (1H, *t*, 11.3 Hz, H-16b)	43.5 (*t*)	3.76 (1H, *dd*, 12.6, 3.6 Hz, H-16a) 3.02 (1H, *dd*, 12.6, 3.6 Hz, H-16b)
17	77.2 (*d*)	3.41 (1H, *dd*, 9.6, 6.3 Hz)	77.0 (*d*)	4.35( 1H, *dd*, 3.6, 8.8 Hz)
18	71.9 (*s*)		150.6 (*s*)	
19	25.5 (*q*)	1.15 (3H, *s*)	111.0 (*t*)	4.96 (1H, *s,* H-19a) 4.77 (1H, *s,* H-19b)
20	25.7 (*q*)	1.17 (3H, *s*)	19.6 (*q*)	1.86 (3H, *s*)
OMe−5	60.7 (*q*)	3.84 (3H, *s*)	62.8 (*q*)	3.96 (3H, *s*)
OH−1		13.73 (1H, *s*)		13.68 (1H, *s*)

^a^ In DMSO-*d_6_*. ^b^ In acetone-*d_6__._*
